# Developmental Programming of Kidney Disease Across the Life Course: A Narrative Review Focused on Inflammation

**DOI:** 10.3390/ijms27052244

**Published:** 2026-02-27

**Authors:** Chien-Ning Hsu, You-Lin Tain

**Affiliations:** 1 Department of Pharmacy, Kaohsiung Municipal Ta-Tung Hospital, Kaohsiung 801, Taiwan; cnhsu@cgmh.org.tw; 2 Department of Pharmacy, Kaohsiung Chang Gung Memorial Hospital, Kaohsiung 833, Taiwan; 3 School of Pharmacy, Kaohsiung Medical University, Kaohsiung 807, Taiwan; 4 Department of Pediatrics, Kaohsiung Chang Gung Memorial Hospital, Kaohsiung 833, Taiwan; 5 Doctoral Program of Clinical and Experimental Medicine, National Sun Yat-Sen University, Kaohsiung 804, Taiwan; 6 College of Medicine, Chang Gung University, Taoyuan 333, Taiwan

**Keywords:** chronic kidney disease, inflammation, cytokines, developmental origins of health and disease (DOHaD), kidney programming, gut microbiota, oxidative stress

## Abstract

Chronic kidney disease (CKD) represents a major global health burden, with growing evidence indicating that its origins extend back to early developmental stages. This narrative review integrates epidemiological, clinical, and mechanistic experimental evidence to position inflammation as a life-course driver of kidney vulnerability rather than a late-stage consequence. Inflammation has emerged as a central mechanistic link connecting adverse prenatal and postnatal exposures to lifelong kidney vulnerability. We highlight the translational potential by identifying pathways amenable to early-life interventions that could modify disease trajectory. During fetal development, maternal nutritional status, metabolic stress, and inflammatory exposures influence nephron endowment, immune maturation, and epigenetic regulation, thereby shaping long-term CKD risk. In childhood, early immune dysregulation and low-grade inflammation contribute to disease initiation, defining critical windows for preventive and renoprotective interventions that can be implemented in at-risk populations. In adulthood and aging, persistent activation of cytokine signaling, inflammasomes, oxidative stress pathways, autophagy–mitophagy imbalance, and cellular senescence drives progressive kidney injury, further amplified by gut microbiota dysbiosis and renin–angiotensin system interactions. Emerging life-course strategies include maternal nutrition optimization, early-life risk stratification, targeted anti-inflammatory and immunomodulatory therapies, and microbiota-directed interventions tailored to developmental stage and individual risk profile. By emphasizing inflammation as a developmentally programmed and preventable process, this review underscores opportunities for early-life and transgenerational CKD prevention, translating mechanistic insights into actionable strategies for preventive medicine and public health.

## 1. Introduction

Chronic systemic inflammation, frequently initiated by oxidative stress and activation of the innate immune system, is increasingly recognized as a central driver of chronic kidney disease (CKD) and its associated cardiovascular complications [1,2,3]. Across the life course, a maladaptive transition from acute, self-limited inflammatory responses to sustained, low-grade inflammation disrupts physiological homeostasis and markedly increases the risk of progressive multi-organ dysfunction [4]. Accordingly, delineating the inflammatory landscape from gestation to senescence is essential for developing integrated and life-course-oriented strategies for kidney disease management [4,5,6,7].

Inflammation exerts a dual role in kidney biology. Precisely regulated inflammatory signaling is indispensable for normal nephrogenesis, coordinating ureteric branching, nephron formation, and renal vascular development [8]. In contrast, excessive or temporally dysregulated inflammation during critical developmental windows compromises nephron endowment and renal architecture [9]. These early-life inflammatory insults induce persistent epigenetic and immune programming, thereby reducing renal functional reserve and increasing susceptibility to hypertension, fibrosis, and CKD later in life [10,11].

Following birth, inflammation acts as a central integrative hub in kidney disease during childhood and adulthood, closely interconnected with oxidative stress, immune dysregulation, aberrant renin–angiotensin system (RAS) activation, and alterations in the gut microbiota [1,2,3,12,13]. Pro-inflammatory signaling intensifies oxidative stress through excessive reactive oxygen species (ROS) generation while concurrently skewing innate and adaptive immune responses toward a chronic, maladaptive state that accelerates renal injury and fibrogenesis. In parallel, gut microbiota dysbiosis amplifies systemic and renal inflammation via microbial metabolites and endotoxins, establishing self-reinforcing inflammatory–metabolic–immune loops that drive kidney disease progression. Notably, the RAS is tightly integrated with inflammatory cascades, forming reciprocal feedback circuits that further exacerbate renal inflammation and structural damage.

With advancing age, inflammaging—defined as chronic, low-grade inflammation associated with immune senescence, cellular stress, and impaired resolution of inflammatory responses—emerges as a key contributor to renal aging [14]. In the kidney, inflammaging promotes endothelial dysfunction, maladaptive immune activation, oxidative stress, and profibrotic signaling, thereby accelerating nephron loss, glomerulosclerosis, and tubulointerstitial fibrosis [15]. Rather than acting as an isolated cause, inflammaging amplifies vulnerability to hypertension and CKD and diminishes renal resilience to additional metabolic or hemodynamic insults.

Given the crucial role of inflammation in kidney disease across the life span, anti-inflammatory therapies have attracted increasing attention as potential CKD treatments [1,15,16]. However, major unmet needs persist, including limited kidney-specific targeting, suboptimal long-term efficacy, and safety concerns related to systemic immunosuppression. Critical gaps also remain in defining disease- and stage-specific inflammatory pathways, identifying reliable biomarkers for patient stratification, and integrating inflammatory mechanisms with metabolic, hemodynamic, and immune processes. Although experimental evidence suggests that early-life modulation of inflammation—particularly during pregnancy or lactation—may prevent adverse kidney programming in offspring, clinical translation remains limited by concerns regarding developmental safety, timing, and off-target effects. These challenges underscore the urgent need for precision, developmentally informed anti-inflammatory strategies that can be safely implemented for kidney disease prevention and management across the entire life course.

**Figure 1:**
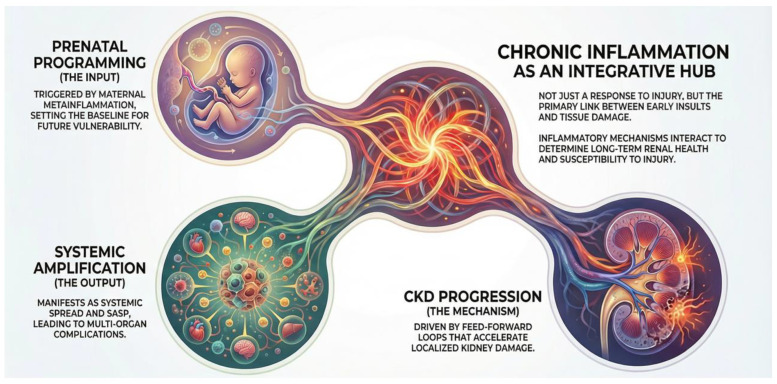
Chronic inflammation as a mechanistic link between early-life kidney vulnerability and chronic kidney disease progression across the life course.

In this narrative review, we highlight the role of inflammation in kidney disease from early development through aging and summarize current evidence supporting strategies to disrupt the vicious cycle of inflammation and preserve renal function throughout the life span (Figure 1).

## 2. Materials and Methods

In light of the wide conceptual scope and substantial variability in study designs across the existing literature, this review was conducted using a narrative framework to facilitate integrative interpretation rather than formal evidence grading. This approach was selected to allow for the synthesis of evolving concepts spanning clinical nephrology, developmental biology, immunology, and therapeutic science. A structured literature search was performed to identify relevant peer-reviewed articles published in English from January 2000 to December 2025. Studies were retrieved from major biomedical databases, including MEDLINE, Embase, and the Cochrane Library, encompassing both experimental models and human investigations. Search terms covered three principal domains: inflammatory mechanisms (e.g., cytokine signaling, inflammasome activation, autophagy, mitophagy, cellular senescence, and innate immune cells), kidney disease and systemic complications (including chronic and end-stage kidney disease, proteinuria, cardiovascular and metabolic disorders), and developmental programming within the DOHaD paradigm (such as maternal exposures, pregnancy, lactation, offspring outcomes, and reprogramming strategies). Bibliographies of selected articles were additionally reviewed to capture studies not identified through database queries. This methodology enabled a cohesive evaluation of mechanistic pathways and translational implications relevant to inflammation-driven kidney programming and CKD progression.

## 3. Early-Life Roots: Fetal Programming and Intrauterine Inflammation

**Figure 2:**
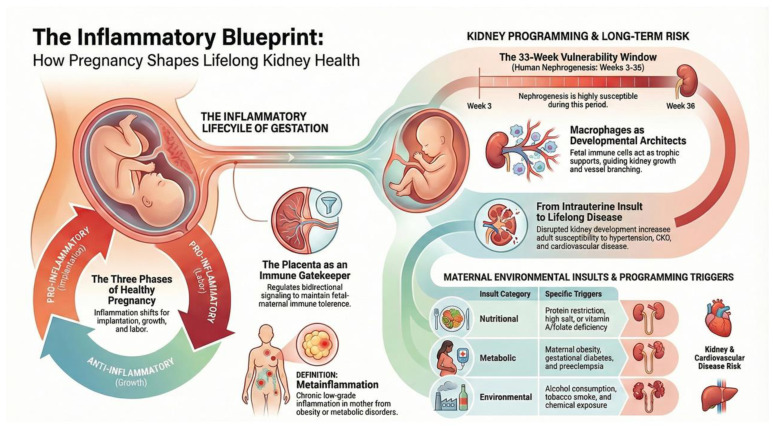
Inflammatory blueprint: Maternal health and prenatal exposures program kidney and immune development via inflammatory pathways, shaping lifelong susceptibility to chronic kidney disease and its related complications.

Figure 2 illustrates the concept of an “inflammatory blueprint”, showing how maternal health and prenatal exposures shape kidney and immune system development, establishing long-term renal risk. Early-life inflammatory signals—driven by cytokines, oxidative stress, and nutrient-sensing pathways—program susceptibility to CKD and its related complications in later life.

### 3.1. Inflammation in Pregnancy and Fetal Development

Inflammation is a core aspect of the innate immune response, essential for host defense and tissue repair. While acute inflammation is tightly regulated and transient, chronic inflammation is sustained and can induce tissue injury and disease progression [17]. During pregnancy, inflammation is not only physiological but essential; however, its magnitude and timing must be precisely controlled to ensure successful gestation and normal fetal development [5].

Early pregnancy is characterized by a localized pro-inflammatory milieu that facilitates embryo implantation and placental establishment. Pro-inflammatory cytokines, including tumor necrosis factor (TNF)-α, interleukin (IL)-1β, IL-6, and IL-8, promote trophoblast invasion, decidual remodeling, and spiral artery transformation, thereby securing adequate oxygen and nutrient delivery to the developing embryo and laying the foundation for organogenesis [18]. As pregnancy progresses into mid-gestation, the maternal immune system shifts toward a predominantly anti-inflammatory and tolerogenic state, which protects the semi-allogeneic fetus and supports rapid fetal growth and placental transport. Approaching term, a controlled reactivation of inflammatory pathways is required to initiate labor and delivery [19].

When inflammatory responses are appropriately orchestrated, they support normal placental function, fetal organ development, and immune programming. In contrast, chronic or excessive inflammation—arising from infection, obesity, metabolic disorders, psychological stress, or autoimmune disease—can disrupt placental signaling and fetal development, increasing the risk of low birth weight, preterm birth, and adverse long-term health outcomes [20]. The placenta serves as a critical interface regulating inflammatory communication between the mother and fetus, underscoring the importance of immune balance throughout gestation [21].

In compromised pregnancies, dysregulation of bidirectional placental signaling—including cytokines, hormones, extracellular vesicles, and immune cell trafficking—can precipitate a breakdown of fetal–maternal immune tolerance. Reduced regulatory T cell activity, inappropriate activation of fetal or maternal effector T cells, and heightened sensitivity to pathogen- and damage-associated molecular pattern (PAMP- and DAMP)-mediated signals collectively amplify inflammatory cascades within the decidua and amniotic cavity. Such premature inflammatory escalation represents a final common pathway linking disrupted fetal–maternal crosstalk to preterm labor [20].

Moreover, maternal metabolic conditions such as obesity and gestational diabetes mellitus induce a state of metainflammation [22], characterized by nutrient excess and sustained elevation of pro-inflammatory cytokines. This inflammatory environment can reprogram fetal gene expression through epigenetic mechanisms, thereby predisposing offspring to CKD and cardiovascular disease (CVD) in later life.

### 3.2. Mechanisms of Developmental Origins

The Developmental Origins of Health and Disease (DOHaD) paradigm posits that the prenatal environment is a critical determinant of lifelong health, linking early-life insults to the later development of many diseases, including CKD [23,24]. Within this framework, kidney programming refers to the disruption of normal nephrogenesis in response to adverse intrauterine conditions [10,11,24].

Although the precise molecular mechanisms remain incompletely defined, substantial progress has been made in identifying key pathways involved in kidney programming. These include oxidative stress, epigenetic modifications, impaired nitric oxide (NO) signaling, aberrant activation of the RAS, epigenetic regulation, altered nutrient-sensing pathways, and gut microbiota dysbiosis [10,11]. Importantly, accumulating evidence indicates that inflammation functions as a central upstream integrator, linking diverse environmental insults to these downstream pathogenic mechanisms.

A wide range of environmental exposures during pregnancy and lactation converge on inflammatory pathways, thereby increasing susceptibility to kidney disease later in life. Both insufficient and excessive maternal nutrient intake can elicit maternal and fetal inflammatory responses [25], as illustrated by epidemiological findings from the Dutch famine birth cohort [26] and studies associating deficiencies in vitamin A [27], folate [28], or overall energy intake [29] with adverse renal outcomes in offspring.

Experimental models further demonstrate that diverse nutritional insults—including protein or calorie restriction [30], vitamin deficiency [31], excessive sugar or fat intake [32,33], and high salt consumption [34]—activate renal inflammatory signaling, leading to impaired nephrogenesis. Maternal metabolic disorders, particularly diabetes and obesity [22], are likewise characterized by chronic low-grade inflammation that interferes with normal kidney development and robustly increases offspring CKD risk. Additional maternal conditions such as hypertensive disorders [35], preeclampsia [36], pre-existing CKD [37], diabetes [38], and systemic inflammation [39] further exacerbate inflammatory milieus during critical windows of renal development in experimental models.

Moreover, maternal exposure to environmental chemicals [40] and substance use, including alcohol [41] and tobacco smoke [42], induces inflammatory and nephrotoxic effects in the developing kidney. Collectively, these findings reinforce inflammation-driven kidney programming as a unifying mechanism underlying increased vulnerability to kidney disease across the life course.

### 3.3. Kidney Programming

**Figure 3:**
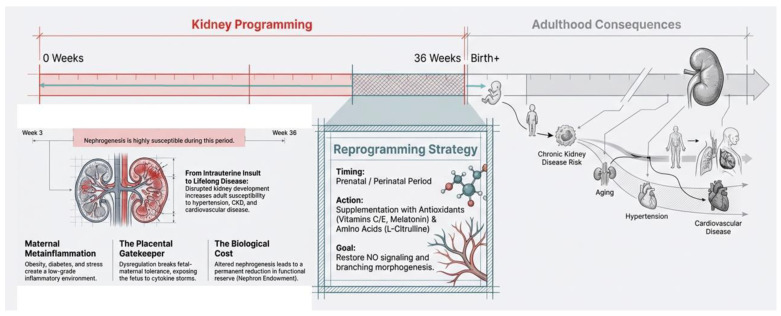
The window of opportunity: Temporal dynamics of kidney programming and reprogramming across developmental stages.

In humans, kidney development begins as early as the third week of gestation and continues until approximately 36 weeks, rendering this process highly vulnerable to perturbations during intrauterine life [43]. Kidney development begins as the ureteric bud extends into the surrounding metanephric mesenchyme. The mesenchyme differentiates into nephrons through a mesenchymal-to-epithelial transition, while coordinated branching of the ureteric bud forms the collecting duct network under precise morphogenetic control [44]. Within this specialized developmental microenvironment, immune cells—particularly macrophages—serve indispensable trophic rather than defensive roles [45,46]. Macrophages colonize the embryonic kidney early in development and actively support renal growth and differentiation through the release of growth factors such as IGF-1, direct cell–cell interactions with nephron progenitor cells, clearance of apoptotic progenitors, and orchestration of ureteric bud branching via VEGF signaling [47]. This finely tuned, low-grade inflammatory milieu is essential for normal nephrogenesis; however, disruption of this balance by adverse intrauterine exposures can shift physiological developmental inflammation toward a maladaptive state, thereby initiating inflammation-driven kidney programming and long-term vulnerability to kidney disease (Figure 3).

High-resolution transcriptomic analyses further reveal dynamic, age-dependent ep-igenetic regulation in the developing kidney, with thousands of differentially expressed genes identified across fetal, neonatal, and juvenile stages [48,49]. Several genes and signaling pathways described in this kidney-developmental framework also have established roles in inflammation, particularly when reactivated or dysregulated after development [50]. WT1 is strongly linked to inflammatory and immune-mediated kidney injury, influencing podocyte inflammation, macrophage behavior, and fibrotic responses [51]. Members of the TGF-β superfamily, especially BMP4 and BMP7, are central regulators of the inflammation–fibrosis axis, with BMP4 tending toward pro-inflammatory effects and BMP7 exerting protective, anti-inflammatory actions in the kidney [52]. VEGF connects angiogenesis with inflammation by promoting endothelial activation and leukocyte recruitment and is closely involved in inflammatory glomerular diseases [53].

Growth factor–receptor pathways critical for ureteric bud development, including GDNF–RET and downstream PI3K/AKT and MAPK signaling, intersect with inflammatory survival and stress pathways [54], while PTEN functions as a key anti-inflammatory brake on PI3K/AKT signaling [55]. Developmental pathways such as Wnt/β-catenin and Notch, although essential for nephrogenesis, are well-recognized drivers of renal inflammation and fibrosis when aberrantly reactivated in postnatal life [56]. FGF signaling also modulates inflammatory repair responses in the kidney.

In contrast, transcription factors such as Pax2/8, Six2, Lhx1, Foxd1, and Tcf21 are primarily developmental regulators but indirectly influence inflammatory susceptibility by shaping nephron progenitor maintenance, stromal cell behavior, and epithelial integrity [57]. Together, these genes form a developmental network that, when perturbed, provides a mechanistic link between abnormal kidney development, maladaptive repair, and chronic renal inflammation.

## 4. Pediatric Considerations: Early Detection and Complications

In the pediatric population, CKD is not merely a localized organ dysfunction but a systemic inflammatory disorder that begins significantly earlier than previously recognized. Evidence indicates that signs of an activated immune system and inflammation can be manifested in pediatric patients with CKD in all stages [58,59]. This persistent, low-grade inflammatory state serves as a pivotal molecular hub, driving a cluster of cardiovascular, renal, and metabolic derangements known as cardiovascular-kidney-metabolic (CKM) syndrome [60]. Unlike the adult population where comorbidities often pre-exist, in children, inflammation frequently functions as the primary trigger for a cascade of complications that resemble a state of “accelerated aging”. Therefore, early identification of systemic inflammation is crucial for risk stratification in children.

### 4.1. Early Detection: Biomarkers and Predictive Value

Chronic low-grade systemic inflammation acts as a central mediator driving coordinated kidney damage, making inflammatory biomarkers critical for early detection, risk stratification, and monitoring disease progression.

In the Chronic Kidney Disease in Children (CKiD) study, several circulating and urinary biomarkers consistently indicate that systemic and intrarenal inflammation is present early in pediatric CKD and independently predicts disease progression [61]. Plasma inflammatory markers—including monocyte chemoattractant protein-1 (MCP-1), TNF receptor-1 and -2 (TNFR-1, TNFR-2), soluble urokinase plasminogen activator receptor (suPAR), and chitinase-3-like protein 1 (YKL-40)—are elevated in children with CKD and show strong, independent associations with faster glomerular filtration rate (GFR) decline after adjustment for proteinuria and serum creatinine. Complementary urinary inflammatory biomarkers, particularly MCP-1 and YKL-40, reflect ongoing intrarenal immune activation and tubulointerstitial inflammation. Together, these findings establish inflammation as a central, early pathophysiologic process in pediatric CKD and support the use of blood and urine inflammatory biomarkers as a “liquid biopsy” to improve risk stratification beyond traditional measures of kidney function.

Classic pro-inflammatory cytokines, including IL-6, TNF-α, and high-sensitivity C-reactive protein (hs-CRP), are closely linked to childhood CKD and its related cardiovascular complications. Elevated IL-6 independently predicts CKD progression and major adverse cardiovascular events, rising as eGFR declines [62]. TNF-α levels are higher in children with CKD versus controls, increase with advancing stage, and are mechanistically linked to inflammatory pathways that contribute to adverse outcomes [63]. hs-CRP is an IL-6–driven acute-phase protein that stratifies cardiovascular risk, even if its independent predictive value for CKD progression varies [64].

Acute-phase reactants such as fibrinogen further indicate systemic inflammation and predict CKD progression [62]. Organ-specific markers provide additional insight: KIM-1 and NGAL detect early diabetic kidney disease and subclinical acute kidney injury [65], and the urinary EGF/MCP-1 ratio predicts histological progression in IgA nephropathy [66]. Endothelial and macrophage markers, including CD163, ICAM-1, and VCAM-1, reflect glomerular inflammation, endothelial activation, and correlate with kidney pathology [67,68].

Metabolic and gut-derived indicators, such as TMAO, indoxyl sulfate, and p-cresyl sulfate, promote oxidative stress, NLRP3 inflammasome activation, and vascular dysfunction [69,70], while elevated leptin contributes to hypertension and glomerulosclerosis [71]. Emerging epigenetic and precision biomarkers, including microRNAs (miR-21, miR-146a) and urinary exosomes, allow for the monitoring of CKD progression and provide information on the cellular origin of inflammatory mediators, guiding personalized interventions [72,73].

Modern multiplex technology allows for the assessment of comprehensive cytokine panels [74]. Pediatric patients often exhibit an imbalanced pattern characterized by elevated pro-inflammatory cytokines such as IL-1, IL-6, and TNF-α, combined with diminished levels of anti-inflammatory mediators like IL-2 and IL-4. This pro-inflammatory signature is inversely correlated with the GFR, becoming more pronounced as renal function declines.

Integrating these biomarkers offers a comprehensive framework to monitor and disrupt the self-perpetuating inflammatory cycle in CKD, enabling the early identification of high-risk or non-responsive patients and facilitating precision therapies to mitigate CKD and its related cardiometabolic complications.

### 4.2. Inflammation as a Central Driver of Complications in Pediatric CKD

Systemic inflammation in pediatric CKD acts as a central pathogenic driver of multi-organ complications rather than a mere secondary phenomenon. Elevated CRP identifies a subgroup at particularly high risk for early vascular aging, arterial calcification, and hypertension. At the molecular level, pro-inflammatory cytokines such as TNF-α and IL-6 promote protein-energy wasting and directly impair growth by suppressing the growth hormone/insulin-like growth factor-1 (GH/IGF-1) axis. In parallel, chronic inflammatory signaling accelerates mineral and bone disorder through the stimulation of osteoclast-mediated bone resorption and disruption of calcium–phosphate homeostasis. Collectively, this persistent inflammatory milieu induces a phenotype of premature or “accelerated” aging in children with CKD, linking growth failure, CVD, and skeletal complications through shared inflammatory mechanisms. Each of these downstream consequences is discussed in detail below.

#### 4.2.1. Hypertension

In pediatric CKD, hypertension is highly prevalent, affecting 50–75% of uremic children [75], and is primarily driven by systemic and intrarenal inflammation. Even in early-stage CKD, elevated pro-inflammatory mediators—including TNF-α, IL-6, MCP-1, suPAR, and YKL-40—impair vascular function [61,62]. ADMA (asymmetric dimethylarginine) inhibits endothelial nitric oxide synthase, reducing NO bioavailability and causing endothelial dysfunction, which promotes oxidative stress, NF-κB activation, and upregulation of pro-inflammatory cytokines, thereby linking ADMA directly to vascular inflammation [76,77]. Adipokines such as leptin further enhance sympathetic nervous system activity, collectively contributing to hypertension [78]. These inflammatory and endothelial pathways form a self-perpetuating cycle in which chronic hypertension drives afferent arteriole remodeling, accelerates glomerular and tubular injury, and reinforces renal inflammation. Consequently, persistent hypertension acts both as a consequence and amplifier of the inflammatory state in pediatric CKD [79,80].

#### 4.2.2. Cardiovascular Disease

CVD is a leading cause of mortality in pediatric CKD, accounting for a substantial proportion of deaths among children initiating dialysis [81]. Chronic inflammation is a central driver of early vascular aging, contributing to left ventricular hypertrophy (LVH), arterial stiffness, and premature vascular remodeling [82]. Cytokines and mediators including IL-6, TNF-α, MCP-1, and suPAR drive endothelial activation, stimulate vascular smooth muscle proliferation, and promote myocardial hypertrophy, collectively accelerating maladaptive cardiac and vascular structural alterations. In parallel, ADMA-mediated NO dysregulation exacerbates endothelial dysfunction and increases vascular tone, while inflammation-induced oxidative stress further impairs arterial compliance [83,84]. Together, these processes link persistent low-grade inflammation to concentric LV remodeling, increased pulse wave velocity, and early atherosclerotic changes, predisposing children with CKD to early-onset cardiovascular morbidity.

#### 4.2.3. Mineral and Bone Disorder

The inflammatory milieu of CKD directly contributes to the development of mineral and bone disorder (CKD-MBD) [85]. Pro-inflammatory cytokines, particularly IL-6 and TNF-α, stimulate RANKL expression, enhancing osteoclast activation and bone resorption [86]. In parallel, elevated MCP-1 and YKL-40 reflect persistent tubulointerstitial inflammation, which disrupts calcium–phosphate homeostasis and accelerates skeletal dysregulation [87]. Vitamin D deficiency frequently coexists with elevated CRP and other acute-phase reactants, further exacerbating abnormalities in bone remodeling and mineral metabolism [88]. This inflammation-driven imbalance underlies the early onset of renal osteodystrophy and increases susceptibility to vascular calcification. Central to this process, Klotho and FGF23 constitute a key inflammatory–mineral regulatory axis in CKD-MBD [89,90]. Inflammatory cytokines upregulate FGF23 production, suppressing calcitriol synthesis, aggravating secondary hyperparathyroidism, and promoting systemic inflammation and cardiovascular injury via Klotho-independent FGFR4 signaling [89,90]. Concurrently, inflammation-mediated Klotho deficiency impairs physiological FGF23 signaling, shifting its effects toward maladaptive pathways that drive disordered bone remodeling, vascular calcification, and progression of CKD-MBD [91].

#### 4.2.4. Impact on Growth and Protein-Energy Wasting

In pediatric CKD, chronic low-grade inflammation is a central driver of growth impairment and protein-energy wasting. Pro-inflammatory cytokines such as TNF-α, IL-6, and IL-1β induce resistance to growth hormone and IGF-1 signaling, suppress chondrocyte proliferation at the growth plate, and impair linear growth [92]. In parallel, inflammation promotes hypercatabolism by activating ubiquitin–proteasome and autophagy pathways, increasing muscle protein breakdown while reducing appetite through cytokine-mediated hypothalamic dysregulation [93]. Systemic inflammation also exacerbates metabolic acidosis, anemia, and insulin resistance, collectively reducing nutrient utilization and anabolic capacity. Together, these inflammation-driven mechanisms contribute to stunted growth, loss of lean body mass, and the development of protein-energy wasting in children with CKD.

#### 4.2.5. Additional Inflammatory Sequelae

Chronic inflammation in pediatric CKD further contributes to anemia and erythropoietin (EPO) resistance. Pro-inflammatory cytokines, including TNF-α, IL-6, and IL-1β, induce hepcidin expression and suppress EPO production, disrupting iron homeostasis [94]. In children receiving peritoneal dialysis, persistent inflammation is associated with increased peritoneal membrane permeability, potentially reducing dialysis efficacy [95]. These multi-system effects demonstrate how sustained inflammation orchestrates diverse complications, encompassing hematologic, metabolic, and cardiovascular systems. Early identification and management of systemic inflammation are critical to mitigating “accelerated aging”, preserving renal function, supporting normal growth, and reducing the long-term burden of CVD in children with CKD.

## 5. Adulthood and Senescence: Inflammaging and Tertiary Structures

**Figure 4:**
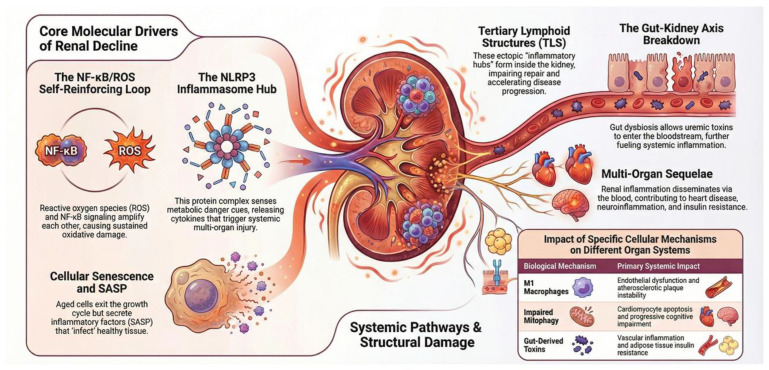
Inflammaging: Biological crosstalk among inflammatory signaling pathways driving chronic kidney disease.

As individuals age, they develop “inflammaging”, a low-grade chronic inflammatory state that promotes fibrosis and accelerates vascular aging. This process is driven by mitochondrial dysfunction, oxidative stress, and the accumulation of uremic toxins. In aged kidneys, persistent inflammation can induce tertiary lymphoid structures (TLSs), ectopic lymphoid aggregates that act as local inflammatory hubs, impairing tissue repair and facilitating CKD progression following acute kidney injury. Senescent cells, which permanently exit the cell cycle but remain metabolically active, secrete a senescence-associated secretory phenotype (SASP) that amplifies both local and systemic inflammation. Understanding these interconnected mechanisms is essential for developing targeted strategies to mitigate inflammaging, preserve renal function, and slow CKD progression in adulthood and senescence. These key processes will be examined in detail in the following sections (Figure 4).

### 5.1. The NLRP3 Inflammasome and Pro-Inflammatory Cytokines

The NLRP3 inflammasome, a cytoplasmic assembly of NLRP3, ASC, and pro-caspase-1, primarily in immune cells, serves as a central mediator of inter-organ inflammation linking CKD and CVD [96]. Its activation requires a priming signal, typically mediated by NF-κB in response to metabolic and inflammatory stimuli such as hyperglycemia and lipotoxicity, followed by an activation signal triggered by cellular danger cues, including potassium efflux, mitochondrial reactive oxygen species, and oxidized mitochondrial DNA [97]. In CKD, uremic toxins such as TMAO, together with metabolic stressors including elevated glucose and free fatty acids, activate NLRP3 inflammasome signaling in immune cells, podocytes, and other renal resident cells, driving renal inflammation and fibrotic remodeling [96,98]. The subsequent release of downstream cytokines—most notably IL-1β and IL-18, along with secondary mediators such as IL-6 and TNF-α—propagates systemic inflammation, metabolic dysregulation, and multi-organ injury [99].

Importantly, several inflammasome-induced cytokines, particularly IL-6 and interferon-related mediators, signal through the JAK/STAT (Janus kinase/signal transducers and activators of transcription) pathway, thereby translating innate immune activation into sustained transcriptional programs that promote chemokine expression, immune cell recruitment, and fibrogenic remodeling [1]. Persistent activation of JAK1/2–STAT3 signaling in renal and vascular tissues amplifies inflammatory cascades and reinforces feed-forward loops linking oxidative stress, endothelial dysfunction, and extracellular matrix deposition.

These cytokine networks establish feed-forward loops that exacerbate oxidative stress, endothelial dysfunction, and tissue fibrosis. Accordingly, therapeutic strategies targeting inflammasome-associated cytokines, including IL-1β and IL-6 inhibition, have emerged as promising approaches to attenuate systemic inflammation and reduce cardiovascular risk in patients with CKD [100].

### 5.2. NF-κB/ROS Loop

Phenomenological evidence from patient samples and mechanistic studies in rodent models indicate that NF-κB is a central transcriptional regulator of inflammatory signaling, controlling the expression of pro-inflammatory cytokines such as IL-1β, IL-6, and TNF-α, as well as key components of the inflammasome machinery [101]. Under physiological conditions, NF-κB remains sequestered in the cytoplasm through its interaction with inhibitory IκB proteins. In CKD, clinical and experimental evidence shows that metabolic and uremic stress—including uremic toxin accumulation, hyperglycemia, dyslipidemia, and excess adiposity—induces IκB phosphorylation and degradation, permitting NF-κB nuclear translocation and sustained transcription of inflammatory mediators that drive renal injury, endothelial dysfunction, and insulin resistance [102]. ROS, primarily generated by dysfunctional mitochondria and NADPH oxidases, act both as downstream effectors and upstream amplifiers of NF-κB signaling. Excessive ROS induce oxidative damage and activate NF-κB through redox-sensitive kinases and danger-associated signals, including oxidized mitochondrial DNA. In turn, NF-κB transcriptionally upregulates ROS-producing enzymes, thereby establishing a self-reinforcing NF-κB/ROS loop that perpetuates chronic inflammation and oxidative stress [103]. This maladaptive loop represents a unifying pathogenic mechanism underlying CKD and its systemic complications. Within the kidney, it promotes podocyte injury, mesangial expansion, and fibrotic remodeling. In the cardiovascular system, it accelerates endothelial dysfunction and atherogenesis. In metabolic tissues, persistent NF-κB/ROS signaling exacerbates insulin resistance and adipose tissue inflammation, while in the central nervous system, it contributes to neuroinflammation and progressive cognitive impairment. Consequently, therapeutic strategies aimed at disrupting this cycle—through the modulation of NF-κB signaling, attenuation of oxidative stress, or downstream cytokine blockade (e.g., IL-1β or IL-6)—represent a rational approach to slowing or potentially reversing multi-organ damage in CKD [104].

### 5.3. Autophagy and Mitophagy

Autophagy is an evolutionarily conserved cellular process essential for maintaining intracellular homeostasis through the lysosomal degradation of damaged organelles, misfolded proteins, and cytoplasmic debris. Mitophagy, a specialized autophagic process, targets and removes damaged mitochondria, helping to restrain reactive oxygen species accumulation and maintain cellular energy balance. In CKD, experimental and observational data show that renal resident cells—including podocytes, tubular epithelial cells, and mesangial cells—depend on intact autophagic and mitophagic flux to cope with nutrient overload, hyperglycemia, lipid accumulation, and uremic toxins [105,106]. Disruption of these pathways causes mitochondrial dysfunction, ROS accumulation, podocyte injury, tubular apoptosis, mesangial expansion, and interstitial fibrosis [107]. Defective mitophagy further amplifies mitochondrial ROS production, which activates NF-κB signaling and NLRP3 inflammasome assembly, establishing a feed-forward loop that perpetuates inflammation, oxidative stress, and glomerular dysfunction in CKD [108].

Beyond the kidney, impaired autophagy and mitophagy contribute to the systemic complications of CKD. In the cardiovascular system, mitochondrial quality-control failure promotes cardiomyocyte apoptosis, endothelial dysfunction, and atherogenesis. Systemically, defective autophagic clearance facilitates the release of mitochondrial DNA and other DAMPs, fueling chronic inflammation that extends to distant organs, including the central nervous system, where it contributes to neuroinflammation and progressive cognitive impairment frequently observed in CKD patients. Restoration of autophagic and mitophagic flux represents a promising therapeutic strategy for mitigating CKD progression and its multi-organ sequelae [109]. Pharmacological modulation using AMPK activators [110], mTOR inhibitors [111], or SIRT1 modulators [112] has been shown to reduce mitochondrial ROS, suppress inflammasome activation, preserve podocyte structure, improve insulin sensitivity, and attenuate vascular inflammation. Collectively, these approaches highlight autophagy and mitophagy as central protective mechanisms and viable intervention targets for achieving multi-organ protection in CKD.

### 5.4. The Gut-Kidney Axis

The gut–kidney axis represents a dynamic bidirectional network linking intestinal microbiota, their metabolites, and renal function, and plays a pivotal role in the pathogenesis of CKD and its systemic complications [113,114]. In CKD, alterations in gut microbiota—marked by loss of microbial diversity and overgrowth of harmful species—disrupt intestinal barrier function, promoting increased permeability and systemic entry of microbial components. Concomitantly, impaired renal clearance results in the accumulation of gut-derived uremic toxins, including TMAO, indoxyl sulfate, and p-cresyl sulfate, which collectively drive inflammation, oxidative stress, and endothelial dysfunction [113,114]. Among these metabolites, TMAO promotes vascular inflammation, platelet hyperreactivity, and atherosclerosis, thereby mechanistically linking microbial dysmetabolism to cardiovascular injury. In parallel, protein-bound uremic toxins such as indoxyl sulfate and p-cresyl sulfate activate NF-κB signaling, NADPH oxidases, and the NLRP3 inflammasome, ultimately accelerating CKD progression [115]. Experimental studies in CKD models further demonstrate that gut dysbiosis skews adaptive immunity toward Th17 and Th1 responses, while increased intestinal permeability facilitates lipopolysaccharide translocation, activating innate immune cells via TLR4- and NF-κB-dependent pathways and amplifying systemic inflammation [116]. Conversely, the microbial fermentation of dietary fiber generates short-chain fatty acids (SCFAs), which exert anti-inflammatory and antioxidative effects and play a crucial role in maintaining intestinal immune homeostasis [117]. SCFAs, particularly butyrate, signal through G protein-coupled receptors expressed in the kidney, where they have been implicated in the regulation of BP and renal inflammation. Loss of SCFA-producing bacteria in CKD therefore removes an important counter-regulatory mechanism, further exacerbating immune dysregulation and metabolic stress. Beyond renal injury, gut-derived uremic toxins also impair insulin signaling in metabolic tissues, aggravating hyperglycemia and adipose tissue inflammation. Together, these processes establish a self-perpetuating feed-forward loop in which kidney dysfunction promotes toxin accumulation, dysbiosis, and barrier disruption, which in turn intensify systemic inflammation and multi-organ damage across the life course [118]. Targeting the gut–kidney axis through dietary fiber supplementation, prebiotics, probiotics, or pharmacologic adsorption of uremic toxins represents a promising therapeutic strategy to attenuate inflammation, modulate microbiota composition, and mitigate CKD progression and its related complications [119].

### 5.5. Cellular Senescence

Cellular senescence is a maladaptive stress response in which cells undergo permanent cell-cycle arrest while maintaining metabolic activity and acquiring a persistent pro-inflammatory state [120]. Mechanistic studies in CKD models demonstrate that persistent metabolic and toxic insults—including hyperglycemia, lipid overload, oxidative stress, and uremic toxins—create a chronic inflammatory milieu that drives the progressive accumulation of senescent cells across renal (podocytes, tubular epithelial cells, mesangial cells) and extra-renal tissues. A defining feature of senescent cells is the SASP, which comprises pro-inflammatory cytokines (e.g., IL-1β, IL-6, TNF-α), chemokines, and matrix-remodeling enzymes, thereby serving as a potent amplifier of local and systemic inflammation [120,121]. Within the kidney, senescence of podocytes, tubular epithelial cells, and mesangial cells directly contributes to glomerulosclerosis, tubular atrophy, and interstitial fibrosis [121]. SASP factors reinforce chronic renal inflammation through sustained activation of NF-κB signaling and inflammasome pathways, establishing a feed-forward loop that perpetuates immune activation, oxidative stress, and progressive nephron loss [122]. Rather than being a passive consequence of injury, senescent renal cells actively reshape the inflammatory microenvironment, accelerating CKD progression. The pro-inflammatory impact of cellular senescence extends beyond the kidney. In the cardiovascular system, senescent endothelial and vascular smooth muscle cells promote endothelial dysfunction, vascular stiffening, and plaque instability [123], thereby heightening cardiovascular risk in CKD. In metabolic tissues, SASP-mediated inflammatory signaling disrupts insulin sensitivity and exacerbates adipose tissue inflammation, reinforcing systemic insulin resistance [124]. Circulating SASP components further disseminate inflammatory cues to distant organs, including the central nervous system, where they may contribute to neuroinflammation and cognitive impairment [125]. Therapeutically, targeting cellular senescence offers a strategy to attenuate inflammation-driven multi-organ damage in CKD [125]. Senolytic interventions that selectively eliminate senescent cells and senomorphic agents that suppress the SASP have been shown to reduce the burden of senescent cells and attenuate pro-inflammatory signaling in preclinical models [125,126,127]. These strategies act on senescent cell survival pathways or downstream inflammatory pathways (e.g., NF-κB, mTOR), interrupting the self-sustaining cycle linking cellular senescence, SASP, and multi-organ dysfunction. Thus, cellular senescence emerges as both a biomarker and a mechanistic driver of inflammation-centered CKD pathophysiology and its systemic complications.

### 5.6. Macrophage

Macrophages are central orchestrators of inflammation and tissue remodeling in CKD and its systemic complications [46]. Due to their remarkable phenotypic plasticity, macrophages dynamically shift along a spectrum from pro-inflammatory M1 to anti-inflammatory and reparative M2 states. In the uremic milieu, chronic metabolic stressors—including hyperglycemia, lipid overload, oxidative stress, and accumulated uremic toxins—skew macrophage polarization toward an M1 phenotype. These M1 macrophages sustain the production of pro-inflammatory cytokines, amplifying inflammatory injury within the kidney and beyond [128]. Within renal tissue, infiltrating and resident M1 macrophages directly contribute to glomerular and tubular damage and serve as key drivers of fibrotic remodeling.

Mechanistic studies demonstrate that a subset of activated macrophages undergoes macrophage-to-myofibroblast transition (MMT), acquiring α-smooth muscle actin expression and adopting a matrix-producing phenotype [129]. MMT-derived myofibroblasts secrete excessive extracellular matrix proteins, accelerating interstitial fibrosis and glomerulosclerosis. This process is tightly regulated by inflammatory and profibrotic signaling pathways, particularly NF-κB and TGF-β/Smad, linking chronic inflammation to irreversible structural damage.

Beyond the kidney, M1-polarized macrophages promote vascular inflammation, en-dothelial dysfunction, and atherosclerotic plaque instability, while driving adipose tissue inflammation and insulin resistance in metabolic organs. Collectively, macro-phage-driven inflammation establishes a self-reinforcing loop of oxidative stress, fibrosis, and multi-organ injury. Therapeutic strategies that reprogram macrophages toward an M2 reparative phenotype or inhibit MMT—through the modulation of TGF-β, NF-κB, or macrophage-targeted interventions—represent a rational approach to attenuating inflammation, limiting fibrosis, and mitigating CKD and its related complications [129,130].

### 5.7. Tertiary Lymphoid Structures

Tertiary lymphoid structures (TLSs) are ectopic lymphoid aggregates that develop in non-lymphoid organs such as the kidney under conditions of chronic inflammation [131]. In CKD, TLSs function as localized inflammatory hubs that directly interact with renal parenchymal cells and integrate metabolic, immune, and aging-related signals. The formation of TLSs is driven by the sustained infiltration and activation of T and B lymphocytes, dendritic cells, and stromal fibroblasts within an inflammatory microenvironment [132]. Immunosenescence contributes critically to TLS expansion: senescence-associated T (SA-T) cells and age-associated B cells accumulate within renal TLSs and interact via the CD153–CD30 axis [133]. SA-T cells secrete pro-inflammatory cytokines such as IFN-γ and IL-21, promoting B-cell activation and TLS maturation. Concurrently, resident fibroblasts produce lymphoid chemokines, including CXCL13 and CCL19, which recruit additional immune cells and sustain chronic inflammation [134]. TLSs exacerbate maladaptive repair and fibrotic remodeling in CKD. TLS-derived cytokines, including TNF-α and IFN-γ, injure VCAM-1-positive proximal tubular cells, which in turn release profibrotic mediators such as TGF-β2 and PDGFB, establishing a self-reinforcing loop of inflammation and fibrosis [132]. In advanced disease, mature TLSs may support local autoantibody production, further amplifying renal injury. Clinically, TLS presence and maturity correlate with accelerated CKD progression and increased risk of end-stage kidney disease, highlighting the CD153–CD30 axis and TLS-associated inflammatory circuits as potential therapeutic targets.

Across adulthood and senescence, chronic inflammation acts as the central organizing principle linking mitochondrial dysfunction, immune dysregulation, cellular senescence, gut dysbiosis, and TLS formation. These interconnected mechanisms converge to amplify renal injury, impair repair, and propagate systemic complications, establishing inflammaging as a unifying driver of CKD pathophysiology and its multi-organ sequelae.

## 6. Anti-Inflammatory Pharmacotherapy and Reprogramming Strategies

**Figure 5:**
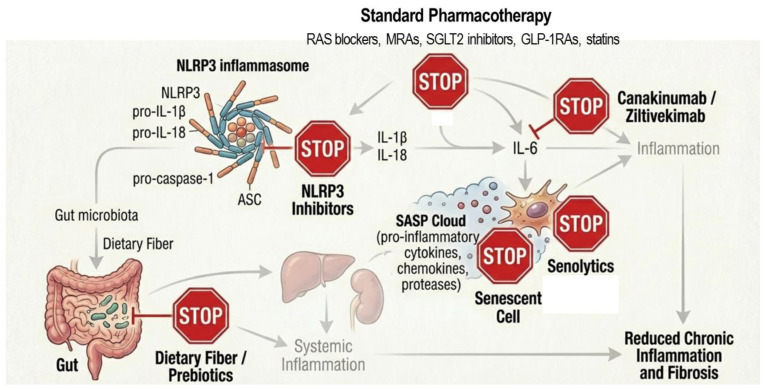
Breaking the inflammatory cycle: From conventional pharmacotherapy to biological reprogramming.

CKD is characterized by persistent low-grade systemic inflammation that orchestrates coordinated injury across multiple organ systems through self-perpetuating cycles of oxidative stress, endothelial dysfunction, and fibrosis, thereby driving disease progression and its associated complications. Accordingly, targeting inflammation has emerged as a central therapeutic strategy for both the treatment and prevention of CKD [1,2,3]. Many standard pharmacotherapies currently used in CKD management exert pleiotropic anti-inflammatory effects beyond their primary indications. In parallel, the development of targeted anti-cytokine and inflammasome-directed therapies offers additional potential to more precisely modulate pathogenic inflammatory pathways [134,135]. Importantly, a paradigm shift is underway toward interventions applied before overt disease onset—so-called reprogramming strategies—which have demonstrated promising renoprotective and cardiometabolic benefits in preclinical models [136]. These therapeutic approaches are discussed in detail below (Figure 5).

### 6.1. Anti-Inflammatory Pleiotropy of Standard Pharmacotherapy

Modern kidney protection strategies increasingly rely on pharmacological agents that confer substantial anti-inflammatory benefits extending beyond their primary metabolic or hemodynamic actions. These pleiotropic effects are now recognized as central contributors to long-term renoprotection and cardiovascular risk reduction in CKD.

#### 6.1.1. RAS Blockade and Nonsteroidal MRAs

RAS inhibitors, including angiotensin-converting enzyme inhibitors (ACEi) and angiotensin receptor blockers (ARBs), remain the cornerstone of CKD therapy by reducing intraglomerular pressure and albuminuria. In vitro studies demonstrate that RAS blockade alleviates proinflammatory stress on proximal tubular cells [137]. Beyond their hemodynamic actions, angiotensin II functions as a proinflammatory cytokine that activates the NF-κB signaling pathway, promoting the expression of inflammatory mediators such as MCP-1 and IL-6 in experimental models [138].

The nonsteroidal mineralocorticoid receptor antagonist finerenone further amplifies anti-inflammatory and antifibrotic protection in preclinical studies by modulating macrophage polarization and suppressing profibrotic gene expression, including plasminogen activator inhibitor-1. Large randomized clinical trials in humans have demonstrated that combination therapy incorporating finerenone, RAS inhibitors, and sodium–glucose cotransporter-2 (SGLT2) inhibitors has demonstrated robust cardiorenal protective effects [139].

#### 6.1.2. SGLT2 Inhibitors

SGLT2 inhibitors have consistently demonstrated efficacy in reducing the onset and progression of renal complications in individuals with and without diabetes in large-scale human outcome trials [140]. A central mechanism involves restoration of tubuloglomerular feedback, whereby increased distal sodium delivery is sensed by macula densa cells, triggering adenosine-mediated afferent arteriolar vasoconstriction and consequent reduction in intraglomerular pressure—a mechanism supported by both physiological studies in humans and experimental models [141]. Additional effects include improvements in tubular oxygenation and energy metabolism, alongside the attenuation of renal inflammation and fibrosis primarily demonstrated in animal and translational studies [142].

At the molecular level, SGLT2 inhibitors suppress activation of the NLRP3 inflammasome and restore cellular quality control through enhanced autophagy and mitophagy in experimental models [143,144]. Agents such as empagliflozin and dapagliflozin reduce oxidative stress and inhibit NF-κB signaling in rodent models and cell-based studies, whereas direct confirmation of these molecular effects in human kidney tissue remains limited. Moreover, SGLT2 inhibition induces a fasting-like metabolic state characterized by activation of the SIRT1/PGC-1α axis in preclinical studies, facilitating organelle turnover and mitigating systemic metainflammation [145].

#### 6.1.3. GLP-1 Receptor Agonists (GLP-1RAs)

Glucagon-like peptide-1 (GLP-1) is a gut-derived incretin hormone released in response to nutrient intake and plays a central role in glucose homeostasis [146]. The development of GLP-1 receptor agonists for the treatment of hyperglycemia has generated increasing interest in their pleiotropic effects, particularly their potential to improve cardiovascular outcomes in diabetic kidney disease, as demonstrated in cardiovascular outcome trials [146].

Beyond glucose lowering, GLP-1RAs—including liraglutide and semaglutide—exert potent immunomodulatory effects by promoting a shift from proinflammatory M1 macrophages toward anti-inflammatory M2 phenotypes through STAT3-dependent signaling [147]. These agents consistently reduce systemic inflammatory biomarkers such as hs-CRP and TNF-α and confer protection against endothelial-to-mesenchymal transition, thereby limiting downstream fibrotic remodeling primarily in experimental models [148].

#### 6.1.4. Statins

Dyslipidemia represents a key modifiable risk factor in CKD, contributing to both cardiovascular morbidity and disease progression. Statin therapy, through the inhibition of HMG-CoA reductase, has become an essential component of CKD management, particularly in advanced stages based on robust human clinical evidence [149]. Importantly, the benefits of statins extend beyond lipid lowering. By inhibiting isoprenoid synthesis, statins modulate small GTPase signaling, improve endothelial function, and reduce circulating levels of CRP and other inflammatory mediators in human studies, while additional anti-inflammatory and antifibrotic mechanisms have been characterized in experimental systems [150,151].

#### 6.1.5. Others

Beyond dedicated kidney-protective therapies, several commonly used metabolic agents exert clinically relevant anti-inflammatory effects that may contribute to renal and vascular protection. Metformin, widely prescribed in patients with diabetes and preserved renal function, activates AMPK, leading to the suppression of NF-κB–mediated inflammatory signaling, reduction in oxidative stress, and improvement of mitochondrial function in cellular and animal models [152,153]. Human data support systemic metabolic benefits, whereas direct intrarenal anti-inflammatory effects in humans remain less well-characterized.

Xanthine oxidase inhibitors, such as allopurinol and febuxostat, reduce the generation of ROS associated with hyperuricemia and inhibit downstream activation of the NLRP3 inflammasome [154]. By lowering uric acid-induced oxidative stress and inflammatory signaling, these agents may attenuate renal and vascular inflammation; however, evidence for slowing CKD progression in randomized human trials remains inconsistent [155].

### 6.2. Targeted Anti-Cytokine and Inflammasome Therapies

Direct pharmacological targeting of discrete inflammatory hubs has emerged as a critical next step in addressing the substantial residual risk that persists in patients with CKD despite optimized standard therapy [1]. Over the past decade, increasing mechanistic insight into CKD-associated immune dysregulation has driven the evaluation of targeted anti-cytokine strategies, including the inhibition of IL-1 and IL-6 signaling, as well as the modulation of upstream inflammatory amplifiers such as the NLRP3 inflammasome. In parallel, agents that enhance endogenous cytoprotective pathways, including Nrf2 activators and therapies aimed at eliminating senescent cells or suppressing SASPs have gained attention. Given its central role in transducing cytokine receptor signaling into sustained pro-inflammatory and pro-fibrotic gene expression, inhibition of the JAK/STAT pathway has emerged as a strategically positioned intervention to dampen multi-organ immune activation and interrupt maladaptive feed-forward inflammatory circuits [1]. Collectively, these approaches represent a shift toward mechanism-driven immunomodulation, with the potential to more precisely attenuate chronic inflammation and slow CKD progression.

#### 6.2.1. Targeting the IL-1/IL-6 Axis

The CANTOS trial provided landmark evidence that the IL-1β monoclonal antibody canakinumab significantly reduces major adverse cardiovascular events in patients with CKD without causing adverse renal events [156]. Other agents like anakinra (IL-1R antagonist) and rilonacept (IL-1 trap) have shown potential in reducing hs-CRP and improving vascular function in advanced CKD [157]. For IL-6, the monoclonal antibody ziltivekimab has demonstrated dose-dependent reductions in inflammatory and atherogenic biomarkers in patients with stage 3–5 CKD in the RESCUE trial [158]. The ongoing ZEUS trial is further evaluating the efficacy of ziltivekimab in reducing MACE and renal endpoints [159].

#### 6.2.2. NLRP3 Inflammasome Inhibition

Direct pharmacological inhibition of the NLRP3 inflammasome has emerged as a promising strategy to mitigate CKD-associated inflammation and progressive tissue injury based predominantly on preclinical CKD models [160]. Small-molecule inhibitors, including MCC950 and AZD4144, selectively block NLRP3 activation, preventing the cleavage of pro–IL-1β and pro–IL-18 by caspase-1 and thereby reducing pyroptotic cell death. By interrupting this central inflammatory cascade, these agents have the potential to attenuate tubular and vascular injury that drives CKD progression.

Additional approaches, such as colchicine, indirectly target NLRP3 signaling. While colchicine demonstrates anti-inflammatory and antifibrotic effects in animal models and cardiovascular outcome trials, evidence specifically supporting renoprotection in CKD remains evolving, and dose adjustment is required in patients with impaired renal clearance [161,162].

#### 6.2.3. Nrf2 Activators and Suppression of SASPs

Activation of Nrf2 represents a key mechanism for restoring cellular redox homeostasis and suppressing pro-inflammatory signaling in experimental CKD models [163]. Nrf2 activators enhance the transcription of antioxidant and cytoprotective genes, reduce oxidative stress, and dampen NF-κB-mediated inflammatory pathways. In the TSUBAKI study, bardoxolone methyl—a synthetic Nrf2 activator—increased measured GFR in carefully selected patients without heart failure risk factors and was generally well-tolerated, supporting Nrf2 activation as a potential strategy to improve renal function while limiting inflammation [164].

Senolytics constitute a complementary approach targeting inflammaging by selectively eliminating senescent cells that accumulate with aging and at pathogenic sites in CKD, as demonstrated in preclinical models [165]. In a phase 1 pilot study of patients with diabetic kidney disease, short-course senolytic therapy with dasatinib plus quercetin significantly reduced senescent cell burden in adipose tissue and skin, accompanied by decreased macrophage infiltration and circulating SASP factors, including IL-1α, IL-6, and MMPs. These findings provide the first direct human evidence that senolytics can reduce senescent cell load, supporting a “hit-and-run” strategy to attenuate SASP-driven inflammation and tissue dysfunction in CKD [166].

### 6.3. Reprogramming Strategies

While anti-inflammatory pharmacotherapies have demonstrated clear benefits in slowing the progression of established CKD, their application as reprogramming strategies before disease onset remains underexplored. Increasing evidence from DOHaD research highlights pregnancy and early life as critical windows during which the targeted modulation of inflammation and immune–metabolic pathways may prevent adverse kidney programming (Figure 2). Accordingly, developing safe, mechanism-based interventions to mitigate inflammaging during gestation represents a promising direction for the primary prevention of CKD in future generations.

#### 6.3.1. Antioxidants

Given that oxidative stress plays a pivotal role in kidney programming and the subsequent development of kidney disease, accumulating evidence indicates that perinatal antioxidant therapy can serve as an effective reprogramming strategy to improve offspring renal outcomes in animal models [167]. These include vitamins C and E [168], amino acids [169,170,171], melatonin [172], resveratrol [173], and N-acetylcysteine (NAC) [174]. In light of the close crosstalk between oxidative stress and inflammation [3], many antioxidants also exert anti-inflammatory effects through redox-sensitive mechanisms. Vitamins C and E attenuate inflammatory responses by scavenging ROS, inhibiting NF-κB activation, suppressing lipid peroxidation, and preserving NO bioavailability [175]. Several amino acids, including L-taurine, L-arginine, and L-citrulline, confer anti-inflammatory benefits primarily through the restoration of NO signaling and attenuation of oxidative stress-induced cytokine production, whereas L-tryptophan and branched-chain amino acids provide indirect immunomodulatory effects, as demonstrated in preclinical models [176]. Melatonin represents a particularly potent antioxidant–anti-inflammatory agent, suppressing NF-κB and iNOS signaling while reducing ADMA, 8-OHdG, and F2-isoprostanes and enhancing NO bioavailability [177]. In addition, resveratrol, synthetic antioxidants, and the thiol donor N-acetylcysteine further mitigate inflammation via the inhibition of NADPH oxidase, activation of SIRT1 or Nrf2 signaling, mitochondrial ROS suppression, glutathione replenishment, and improvement of endothelial function [178,179]. Collectively, these antioxidants link oxidative stress modulation to anti-inflammatory protection in kidney programming based predominantly on preclinical models.

#### 6.3.2. Inhibition of NLRP3 and NF-κB

Maternal obesity activates the NLRP3 inflammasome in the fetal rat kidney, driven by peroxisome depletion-associated oxidative stress and ER stress, leading to pro-inflammatory cytokine release and programmed susceptibility to hypertension and kidney disease in adult rat offspring [180]. Beyond the kidney, maternal high-fat diet exposure also induces NLRP3/IL-1β-dependent vascular inflammation in rat offspring, characterized by endothelial dysfunction, reduced NO bioavailability, and elevated BP, all of which are reversible by NLRP3 or IL-1β inhibition [181]. These inflammasome-driven responses are accompanied by disrupted antioxidant defenses and maladaptive UPR signaling in fetal tubular epithelial cells. Notably, the antioxidant pyrroloquinoline quinone (PQQ) suppresses ROS accumulation and functionally attenuates NLRP3 inflammasome activation [180], although whether direct NLRP3 inhibition is sufficient to protect against renal developmental programming remains to be determined.

Prenatal exposure to the inflammatory stimulus zymosan induces long-term hypertension in rat offspring, associated with sustained NF-κB activation in the kidneys. Gestational inhibition of NF-κB with pyrrolidine dithiocarbamate (PDTC) prevents both NF-κB activation and the programmed rise in BP in animal models [182]. Similarly, perinatal NF-κB inhibition with PDTC in fawn-hooded hypertensive rats normalizes early renal NF-κB activity, reduces blood pressure, proteinuria, and glomerulosclerosis [183]. These findings highlight the potential of NF-κB-targeted interventions in kidney programming.

#### 6.3.3. Nrf2 Activation and Autophagy

Maternal interventions can modulate offspring kidney inflammation and autophagy, thereby influencing long-term renal outcomes in preclinical models. Dimethyl fumarate, a potent Nrf2 activator, enhances autophagy and protects male rat offspring from hypertension programmed by prenatal dexamethasone exposure combined with a postnatal high-fat diet [184]. Similarly, maternal quercetin intake during lactation restores autophagic flux and attenuates renal inflammation—characterized by reduced macrophage infiltration and pro-inflammatory cytokine expression—in adult female rat offspring exposed to a high-fructose diet [185]. Collectively, these findings underscore autophagy enhancement and anti-inflammatory actions as central mechanisms in renal reprogramming.

Activated AMP-activated protein kinase (AMPK) promotes autophagy by directly phosphorylating autophagy-related proteins within the mTORC1, ULK1, and PIK3C3/VPS34 complexes, regulating autophagy-related gene expression via transcription factors such as FOXO3, TFEB, and BRD4, and facilitating mitophagy through the clearance of damaged mitochondria [186]. Emerging evidence supports targeting AMPK activators, including polyphenols and metformin, as effective reprogramming strategies to prevent adverse renal outcomes induced by kidney programming [187,188]. Various classes of polyphenols—such as flavonols [185], flavanols [189], lignans [190], and stilbenes [173]—have demonstrated renoprotective effects in experimental models of kidney programming. Beyond dietary compounds, prenatal metformin exposure has been shown to prevent hypertension programmed by a combined maternal high-fructose intake and post-weaning high-fat diet, an effect linked to AMPK activation and the attenuation of oxidative stress [191]. These observations suggest that AMPK activation during critical developmental windows exerts sustained reprogramming effects, likely through coordinated modulation of autophagy and redox homeostasis, ultimately benefiting offspring renal health in preclinical models.

#### 6.3.4. Senolytics and the SASP

Emerging evidence further links the developmental programming of hypertension to chronic inflammatory signaling. Maternal high-salt intake induces the long-term elevation of blood pressure in male rat offspring by activating c-Src/MAPK/NF-κB-dependent inflammation in the paraventricular nucleus, leading to increased sympathetic activity and disruption of excitatory–inhibitory neurotransmitter balance [192]. Notably, central infusion of the c-Src inhibitor dasatinib attenuates this inflammatory cascade and normalizes neurotransmitter homeostasis, highlighting how targeting senescence- and SASP-like inflammatory signaling pathways may mitigate programmed cardiovascular and neuroinflammatory dysfunction.

#### 6.3.5. Gut Microbiota and Derived Metabolites

The gut–kidney axis provides a systemic pathway through which early-life interventions can reprogram host inflammatory and metabolic states [13]. In experimental and human CKD, gut dysbiosis promotes the accumulation of microbiota-derived uremic toxins, such as indoxyl sulfate and TMAO, while reducing the production of SCFAs. SCFAs, including acetate and butyrate, function as natural histone deacetylase inhibitors and ligands for G protein-coupled receptors, thereby restoring intestinal barrier integrity, promoting regulatory T-cell expansion, and suppressing NF-κB and NLRP3 inflammasome signaling to mitigate systemic inflammation [193,194].

In addition, gut microbiota-derived tryptophan metabolites act as both uremic toxins and aryl hydrocarbon receptor (AhR) ligands, mechanistically linking dysbiosis to oxidative stress, endothelial dysfunction, renal inflammation, and hypertension. Experimental studies demonstrate that maternal CKD-induced rat offspring hypertension is associated with altered tryptophan-metabolizing microbes, AhR activation, and TH17-driven renal inflammation [195]. Targeting this pathway through maternal tryptophan supplementation [195] or perinatal administration of AhR modulators, such as resveratrol [196], restores microbial composition and AhR signaling, thereby preventing hypertension of developmental origins.

Beyond tryptophan metabolism, maternal inhibition of TMAO generation using 3,3-dimethyl-1-butanol [197] or iodomethylcholine [198] has been shown to protect adult rat offspring from hypertension following prenatal insults. Similarly, in rodent models, maternal high-fructose intake programs hypertension in offspring, an effect that can be prevented by perinatal supplementation with the SCFAs butyrate or propionate in experimental studies [199]. These protective effects are mediated through the modulation of gut microbiota composition and metabolites, with butyrate conferring additional benefits on TMAO metabolism. Collectively, these findings highlight microbiota-derived metabolites as actionable reprogramming targets for preventing kidney programming-related hypertension.

### 6.4. Current Gaps and Future Directions

Anti-inflammatory therapy in kidney disease has evolved from early-phase safety assessments to large-scale outcome trials [1]; however, its clinical translation remains limited by several unresolved challenges. Importantly, much of the mechanistic rationale for these interventions is derived from in vitro experiments and animal models, whereas robust evidence from randomized controlled trials in humans remains comparatively limited. A major concern is safety, as systemic cytokine inhibition and upstream pathway modulation may increase susceptibility to infections or induce sodium retention and heart failure, thereby offsetting potential renal benefits [100]. These risks are further exacerbated by the uremic paradox [200], in which patients with advanced CKD exhibit concurrent immune activation and immune suppression, rendering broad immunomodulatory strategies inherently hazardous [201].

Another critical gap arises from marked biological heterogeneity and functional redundancy within inflammatory pathways. Cytokines such as IL-6 display pleiotropic and context-dependent actions, complicating therapeutic target selection and contributing to inconsistent clinical trial outcomes [202]. Moreover, while numerous agents demonstrate efficacy in experimental kidney injury or CKD models, these benefits have frequently not translated into meaningful clinical endpoints in human studies, underscoring persistent translational discrepancies driven by species-specific immune responses, differences in disease chronicity, comorbidities, and the limited ability of current animal models to fully recapitulate the complexity of human kidney disease [203]. Despite advances in standard-of-care therapies, a substantial residual inflammatory risk persists, highlighting the urgent need for improved patient stratification and predictive biomarkers to identify individuals most likely to benefit from adjunctive anti-inflammatory interventions.

Ongoing clinical trials targeting IL-6 signaling, the NLRP3 inflammasome, Nrf2 activation, cellular senescence, complement pathways, and interferon-mediated injury reflect a shift toward more mechanism-informed therapeutic strategies [1]. However, it should be emphasized that for several of these targets, human data are preliminary, and long-term safety and efficacy remain to be established. Future progress is likely to depend less on single-pathway inhibition and more on precision approaches that integrate immune phenotyping, biomarker-guided patient selection, and rational combination therapies [204,205,206]. Bridging these gaps is essential for translating anti-inflammatory strategies into safe, durable, and clinically meaningful treatments for kidney disease.

Importantly, evidence supporting inflammation-targeted reprogramming strategies in kidney programming is derived almost exclusively from preclinical studies. To prevent overinterpretation, we explicitly acknowledge that therapeutic implications in this context remain hypothetical and experimental. Although animal models represent a necessary intermediate step between bench research and human trials and are a regulatory requirement for validating early experimental findings [207], further studies are required to define the optimal timing, dosage, and duration of inflammation-based interventions across different models of kidney programming, as well as to establish translatability in well-designed human cohorts and clinical trials before successful clinical application can be achieved.

## 7. Conclusions

The transition toward precision nephrology requires defining individual inflammatory phenotypes to guide personalized therapeutic strategies. Advances in omics technologies enable the identification of biomarkers associated with suboptimal responses to standard care, thereby facilitating the rational integration of targeted anti-cytokine biologics, epigenetic modulators, and senotherapeutic agents. Addressing residual inflammatory risk from the earliest stages of kidney programming through reprogramming-based interventions represents a promising avenue to reduce the global burden of CKD. Ultimately, effective kidney disease management will depend on shifting from nonspecific immunosuppression to targeted immunomodulation and biological reprogramming, integrating conventional pharmacotherapy with mechanism-based interventions to disrupt the inflammatory cycle and preserve kidney function across the life course.

## Data Availability

No new data were created or analyzed in this study. Data sharing is not applicable to this review article.
